# Evaluation of the correlation of KAI1/CD82, CD44, MMP7 and β-catenin in the prediction of prognosis and metastasis in colorectal carcinoma

**DOI:** 10.1186/s13000-015-0411-0

**Published:** 2015-09-25

**Authors:** Qiong Wu, Yan Yang, Shiwu Wu, Wanyun Li, Na Zhang, Xiuqin Dong, Yurong Ou

**Affiliations:** Department of Pathology, The First Affiliated Hospital of Bengbu Medical College, Bengbu Medical College, Bengbu, Anhui 233004 China; Department of Medical Oncology, The First Affiliated Hospital of Bengbu Medical College, Bengbu, Anhui 233004 China

**Keywords:** CRC, Metastasis, Prognosis, β-catenin, KAI1/CD82, CD44, MMP7

## Abstract

**Background:**

To investigate the relationship of KAI1/CD82, CD44, matrix metalloproteinase 7 (MMP7) and β-catenin, and examine its association with clinicopathological features, metastasis and prognosis in colorectal carcinoma (CRC).

**Methods:**

Immunohistochemical (IHC) analysis was used to detect the expression of KAI1/CD82, CD44, MMP7 and β-catenin in 174 archival surgical specimens of human CRC. Furthermore, clinicopathological features such as age, sex and so on were also collected retrospectively.

**Results:**

CD44, MMP7 and β-catenin expression was positively associated with distant metastasis, lymph node metastasis and tumor-node-metastasis (TNM) stage. However, decreased KAI1/CD82 expression correlated significantly with distant metastasis, lymph node metastasis and TNM stage. KAI1/CD82 expression showed a negative correlation with CD44, MMP7 and β-catenin. Furthermore, β-catenin expression showed a positive correlation with CD44 and MMP7. Multivariate logistic regression analysis showed that KAI1/CD82 and β-catenin expression were significantly associated with lymph node metastasis and KAI1/CD82 was significantly associated with distant metastasis. Kaplan-Meier analysis revealed that CD44, MMP7 and β-catenin expression was negatively correlated with overall survival (OS), while KAI1/CD82 expression was positively correlated with OS. Low KAI1/CD82 expression and high expression of CD44, MMP7 and β-catenin was associated with a poor prognosis in CRC. Multivariate Cox regression analysis indicated that the expression of KAI1/CD82, MMP7 and β-catenin were independent predictors of OS in CRC.

**Conclusion:**

The expression of KAI1/CD82, CD44, MMP7 and β-catenin is related to tumor metastasis and prognosis in CRC. Combined detection of these factors may be of significant value in predicting the prognosis and metastasis in CRC patients.

## Background

In recent years, along with changes in lifestyle, the incidence of CRC has increased rapidly to become the fifth most commonly diagnosed cancer in China [1]. Although the incidence in the United States has decreased significantly due to improved treatments as well as increased awareness and early screening [2], CRC remains the third leading cause of cancer deaths in both men and women [3]. Metastasis in CRC is a major factor responsible for poor prognosis [4]; therefore, the identification of novel molecular markers of a metastatic phenotype is a major challenge in CRC therapy [4, 5].

The KAI1/CD82 protein is a member of the TM4SF (transmembrane 4 superfamily), which mediates signal transduction both between cells and between cells and the extracellular matrix (ECM) [6]. KAI1/CD82 was originally identified as a suppressor of metastasis located on human chromosome 11p11.2 in prostate carcinoma [7]. The majority of evidence indicates that KAI1/CD82 expression is downregulated or abolished in a variety of malignant tumors [8].

CD44 is an extensively expressed class I transmembrane glycoprotein distributed on many normal cells and tumor cells [9]. CD44 acts initially as an adhesion factor that mediates cell-cell and cell-matrix interactions [10]. In the most well-known interaction, CD44 acts as a cell surface receptor for HA (hyaluronic acid), which is closely related to the invasion and metastasis of tumor cells [11].

MMP7, also known as matrilysin, is the minimum structure of the MMP family, which has a broad substrate specificity for ECM components, including elastin, gelatin, type IV collagen, fibronectin, and laminin [12]. MMP7 is known to be overexpressed in a variety of malignant tumors and plays an important role in metastasis [13].

β-catenin forms a complex with cadherin on the cell membrane, forming links to the cytoskeleton that are essential for the cell-cell adhesion [14]. Furthermore, β-catenin is an essential cytoplasmic signal transducer of the canonical Wnt signaling pathway. When the pathway is activated, cytoplasmic β-catenin is transferred into the nucleus, where it combines with transcription factors of the TCF/LEF family to modulate target genes [15]. β-catenin is frequently found to be mutated in virtually all intestinal cancers resulting in activation of the Wnt/β-catenin pathway [16]. Moreover, studies suggest that β-catenin overexpression in the nucleus and cytoplasm is closely related to metastasis and the prognosis in CRC [17, 18].

Overall, studies of KAI1/CD82, CD44, MMP7 and β-catenin in relation to tumor metastasis indicate that these molecules are involved in the process of tumor progression through regulating the intercellular adhesion [6, 11, 12, 14]; However, there are few studies on the interaction between them. In this study, we investigated the hypothesis that there is a mutual relationship between these factors and the interaction of these factors is related to metastasis and prognosis in CRC.

## Methods

### Patients and tissue samples

All 174 CRC tissues and surrounding “normal” mucosa tissues were collected from the Department of Pathology, at the First Hospital Affiliated to Bengbu Medical College, (China) from January 2005 to December 2006. Patients underwent radical resection and peripheral mesenteric lymph node dissection. The “normal” mucosa tissues were removed from the same patient, avoiding necrotic tissues, and from surrounding mucosa at least 3 cm away from the tumor edge. All patients were sporadic cases who had complete clinical, pathological and follow-up data, and no history of hereditary CRC. We excluded patients who received preoperative chemotherapy or radiotherapy. All patients were followed-up at 6-month intervals by phone, mail, or email. Survival time was calculated from surgery to death; data from patients who died from disease unrelated to CRC, accident and those who were lost to follow-up at December 2013 were censored (mean survival time: 51.78 months; range 8–108 months). Tumor differentiation grade was defined according to World Health Organization criteria. Clinical stages were defined according to International Union Against Cancer/American Joint Committee on Cancer TNM criteria. The age of the patients ranged from 23 to 80 years (median age, 62.1 years). Other clinicopathogical characteristics are provided in Table 1.

**Table 1:** Patient characteristics

Patient characteristic	Frequency (*n*)	Percentage (%)
Sex		
Male	101	58.0
Female	73	42.0
Age		
60 years	68	39.0
≥ 60 years	106	61.0
Diameter of tumor		
5.0 cm	104	59.8
≥ 5.0 cm	70	40.2
Location		
Rectum	89	51.1
Colon	85	48.9
Differentiation		
Well	39	22.4
Moderate	90	51.7
Poor	45	25.9
Depth of invasion		
Under serous membrane	96	55.2
To serous membrane	78	44.8
Lymph node metastasis		
Negative	103	59.2
Positive	71	40.8
Distant metastasis		
Negative	150	86.2
Positive	24	13.8
TNM stage		
I + II	101	58.0
III + IV	73	42.0

This study was approved by Ethics Committee of the First Hospital Affiliated of Bengbu Medical College and conducted in accordance with the ethical guidelines of the Declaration of Helsinki.

### Immunohistochemical analysis

All specimens were fixed in 10 % buffered formalin, embedded in paraffin and sectioned (thickness, 4 μm). Sections were then deparaffinized and rehydrated with xylene and graded alcohol. Subsequently, the sections were washed in phosphate-buffered saline (PBS, pH 7.2) for 10 min. The endogenous peroxidase activity was blocked by incubation in 3 % H_2_O_2_ at room temperature for 10 min, then heated to 95 °C for 30 min for antigen retrieval. After washing in PBS three times, the sections were blocked in goat serum and incubated with KAI1/1CD82 (clone H-173, dilution 1:200, Santa Cruz Biotechnology, CA, USA),CD44 (clone DF1485, dilution1:200, Santa Cruz Biotechnology, CA, USA ), MMP7 (clone L-17, dilution1:150, Santa Cruz Biotechnology, CA, USA ), and β-catenin (clone C-18, dilution 1:200, Santa Cruz Biotechnology, CA, USA) primary antibodies at 4 °C overnight. Subsequently, the slides were incubated with polymer enhancer (reagent A), goat anti-mouse antibody (reagent B) and developed in freshly prepared 3,3'-diaminobenzidine (DAB) substrate. Finally, sections were counterstained with hematoxylin, dehydrated, air-dried, and mounted.

### Evaluation of immunostaining

All slides were evaluated by two experienced pathologists who were blinded to the clinical data or the disease outcome. The immunostaining was determined in 10 fields (×100 magnification) for each slide. To evaluate KAI1/CD82, CD44 and MMP7 expression, the staining of entire carcinoma-involved area was graded in terms of both extent and intensity [19]. The intensity of the staining was divided into four grades: 0, none; 1, weak; 2, moderate; 3, strong. The extent of staining was also divided into five categories: 0, ≤5 %; 1, 6–25 %; 2, 26–50 %; 3, 51–75 %; 4, 76–100 %. Finally, we determined the score by multiplying the intensity and the extent of staining to produce a range of immunostaining scores from 0 to 12. The immunostaining was considered positive when the scores were ≥3.

Intracellular brown particles were deemed as positive for β-catenin, and positive β-catenin located at the cell membrane, cytoplasm and nucleus. The results were determined according to the method of Maruyama et al. [18] Normal expression was defined as positive membrane staining seen in >70 % cells, otherwise, it was deemed as a deletion of membrane expression. Positive cytoplasmic and nuclear expression was defined when staining was observed in >10 % cells. Deletion of membrane expression and positive cytoplasmic and nuclear expression were proposed as defined abnormal expression.

### Statistical analysis

Statistical analysis was performed using SPSS 20.0 software for windows (New York, IBM, USA). Fisher's exact or Pearson Chi-square tests were used to analyze the relationship between protein expression and clinicopathogical indices. Univariate analysis to compare distant metastasis, lymph node metastasis and clinicopathogical indices was performed using Fisher's exact or Pearson Chi-square tests. Correlations between the expression of these factors were evaluated by Spearman’s correlate analysis. Multivariate logistic regression analysis was used to clarify the relative factors for metastasis. OS was defined as the time from surgery to death or the end of follow-up. The univariate survival analysis of OS was based on the Kaplan–Meier method with log-rank tests. A multivariate Cox regression model was used to analyze the influence of various factors on OS. Covariates consisted of sex, age, tumor diameter, location, differentiation, depth of invasion, lymph node metastasis, distant metastasis, and expression of KAI1/CD82, CD44, MMP7 and β-catenin. Beta coefficients and 95 % confidence intervals (CI) were used for analysis. A value of *P* < 0.05 was considered to indicate statistical significance.

## Results

### Expression of KAI/CD82, CD44, MMP7 and β-catenin in malignant and normal tissues

**Fig. 1:**
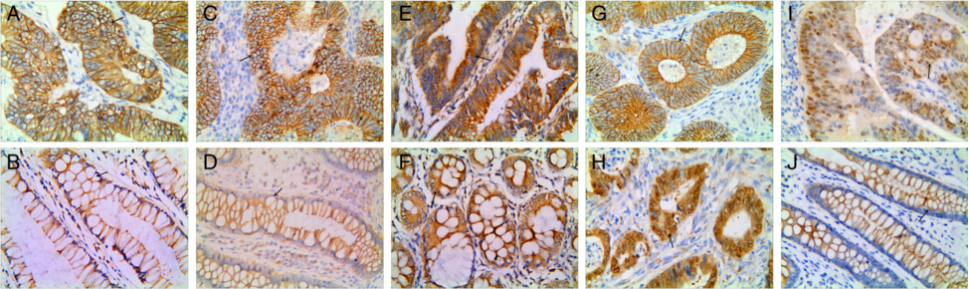
Expression of the proteins in colorectal carcinoma (×400 magnification). **a** Positive KAI1/CD82 expression in the membrane of cancer cells (*arrow*). **b** Positive KAI1/CD82 expression in the membrane of “normal” mucosa cells (*arrow*). **c** Positive CD44 expression in the membrane of cancer cells (*arrow*). **d** Positive CD44 expression in the membrane of “normal” mucosa cells (*arrow*). **e** Positive MMP7 expression in the membrane and cytoplasm of cancer cells (*arrow*). **f** Positive MMP7 expression in the membrane and cytoplasm of “normal” mucosa cells (*arrow*). **g** Positive β-catenin expression in the membrane of cancer cells (*arrow*). **h** Positive β-catenin expression in the nucleus and cytoplasm of cancer cells (*arrow*). **i** Positive β-catenin expression in the nucleus of cancer cells (*arrow*). **j** Positive β-catenin expression in the membrane of “normal” mucosa cells (*arrow*)

In present study, positive KAI1/CD82 expression was detected on the membrane of CRC and “normal” mucosa cells [20] (Fig. 1a and b)**.** KAI1/CD82 protein was expressed positively in 32.1 % (56/174) of CRC and 54.6 % (95/174) of “normal” mucosa tissues. Positive CD44 expression was detected on the membrane of CRC and “normal” mucosa cells [5] (Fig. 1c and d). CD44 protein was expressed positively in 60.9 % (106/174) of CRC and 27.6 % (48/174) of “normal” mucosa tissues. Positive MMP7 expression was detected on the cytoplasm and membrane of CRC and “normal” mucosa cells [21] (Fig. 1e and f). MMP7 protein was expressed positively in 64.9 % (113/174) of CRC and 15.5 % (27/174) of “normal” mucosa tissues. The percentage of positive KAI1/CD82, CD44 and MMP7 were different between “normal” mucosa and CRC (*P* < 0.05) (date not shown); In our study, β-catenin expression was detected on the membrane or nucleus and on the cytoplasm of the CRC cells, although we considered the mutant expression (in the nucleus and cytoplasm) as positive expression [17, 18] (Fig. 1g,h and i) and β-catenin expression was detected in 129 (74.1 %) specimens. In addition, the majority of β-catenin expression was detected on the cell membrane of “normal” mucosa tissues (Fig. 1j), only 2.9 % (5/174) expression was considered to be mutant expression.

### Correlation between KAI1/CD82, CD44, MMP7, β-catenin expression levels and clinicopathological characteristics

There was no relationship between KAI1/CD82, CD44, MMP7, β-catenin expression and sex, age, tumor diameter, and location (*P* > 0.05). The expression of CD44, MMP7 and β-catenin showed a positive correlation with TNM stage, distant metastasis and lymph node metastasis (*P* < 0.05). The expression of KAI1/CD82 showed a negative correlation with differentiation, depth of invasion, TNM stage, distant metastasis and lymph node metastasis (*P* < 0.05) (Table 2).

**Table 2:** The relationship between expression of KAI1/CD82, CD44, MMP7 and β-catenin and clinicopathogical characteristics of colorectal carcinoma (CRC)

Variables	β-catenin expression		*P*	KAI1/CD82 expression		*P*	CD44 expression		*P*	MMP7 expression		*P*
	Negative	Positive		Negative	Positive		Negative	Positive		Negative	Positive	
Sex			0.966			0.871			0.882			0.274
Male	26	75		68	33		39	62		39	62	
Female	19	54		50	23		29	44		22	51	
Age			0.392			0.531			0.855			0.985
60 years	20	48		48	20		26	42		24	44	
≥ 60 years	25	81		70	36		42	64		37	69	
Diameter of tumor			0.752			0.861			0.838			0.665
5.0 cm	26	78		70	34		40	64		38	66	
≥ 5.0 cm		51			22			42			47	
Location			0.164			0.908			0.387			0.182
Rectum	19	70		60	29		32	57		27	62	
Colon	26	59		58	27		36	49		34	51	
Differentiation			0.731			0.002			0.157			0.852
Well	11	28		18	21		20	19		15	24	
Moderate	21	69		63	27		30	60		30	60	
Poor	13	32		37	8		18	27		16	29	
Depth of invasion			0.449			0.008			0.087			0.694
Under serous membrane	27	69		57	39		43	53		35	61	
To serous membrane	18	60		61	17		25	53		26	52	
Lymph node metastasis			0.001			<0.001			0.002			0.026
Negative	36	67		58	45		50	53		43	60	
Positive	9	62		60	11		18	53		18	53	
Distant metastasis			0.009			0.007			0.015			0.042
Negative	44	106		96	54		64	86		57	93	
Positive	1	23		22	2		4	20		4	20	
TNM stage			0.016			<0.001			<0.001			<0.001
I + II	33	68		52	49		52	49		48	53	
III + IV	12	61		66	7		16	57		13	60	

### Correlations among KAI1/CD82, CD44, MMP7 and β-catenin in CRC

There was a negative correlation between KAI1/CD82 expression and expression of CD44, MMP7, and β-catenin expression (*r* = -0.381; *r* = -0.448; *r* = -0.267, respectively; *P* < 0.001) (Table 3). The expression of β-catenin showed a positive correlation with CD44 and MMP7 expression (*r* = 0.199, *P* = 0.008; *r* = 0.226, *P* = 0.003). The expression of CD44 and MMP7 showed a positive correlation (*r* = 0.339, *P* < 0.001) (Table 3).

**Table 3:** Correlation between expression of KAI/CD82, CD44, MMP7 and β-catenin in CRC

Variables	KAI1/CD82		*r*	*p*	CD44		*r*	*p*	MMP7		*r*	*p*
	negative	positive			negative	positive			negative	positive		
β-catenin			−0.239	0.001			0.199	0.008			0.226	0.003
Negative	22	23			25	20			24	21		
Positive	96	33			43	86			37	92		
KAI1/CD82							−0.406	<0.001			−0.474	<0.001
Negative					30	88			23	95		
Positive					38	18			38	18		
CD44											0.399	<0.001
Negative									40	28		
Positive									21	85		

### Metastasis analysis

Univariate analysis showed that tumor invasion correlated positively with distant metastasis and lymph node metastasis (*P* < 0.05) (date not shown). In the multivariate logistic regression analysis, depth of invasion, KAI1/CD82 and β-catenin were significantly associated with lymph node metastasis, while KAI1/CD82 was significant associations with distant metastasis (Table 4).

**Table 4:** Multivariate analysis of factors affecting lymph node metastasis and Distant metastasis

Variables	Categories	Multivariate analysis		
		HR	95 % CI	*p*
Lymph node metastasis				
Depth of invasion	Under serous membrane/To serous membrane	4.326	2.166-8.639	<0.001
KAI1/CD82	Negative/positive	0.325	0.144-0.732	0.007
β-catenin	Negative/positive	3.426	1.404-8.358	0.007
Distant metastasis				
KAI1/CD82	Negative/positive	0.206	0.046-0.925	0.039

### Survival analysis

**Fig. 2:**
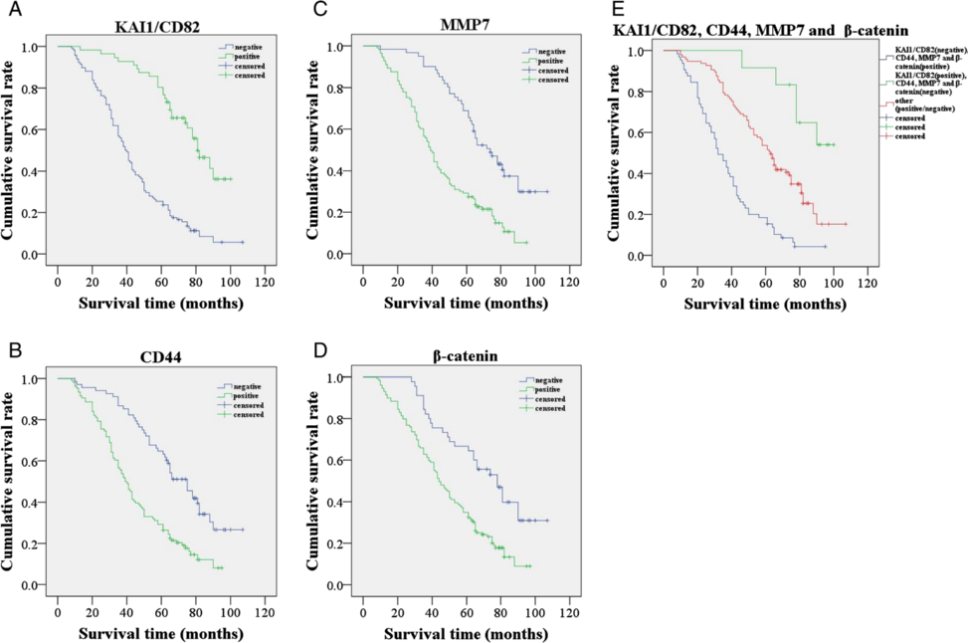
Kaplan–Meier analysis of the survival rate of patients with colorectal carcinoma. **a** Overall survival of all patients in relation to KAI1/CD82 expression (log-rank =46.961, *P* < 0.001). **b** Overall survival of all patients in relation to CD44 expression (log-rank = 24.611, *P* < 0.001). **c** Overall survival of all patients in relation to MMP7 expression (log-rank = 27.764, *P* < 0.001). D Overall survival of all patients in relation to β-catenin expression (log-rank = 15.756, *P* < 0.001). In **a**, **b**, **c** and **d** analyses, the green line represents positive expression of proteins and the blue line represents negative expression of proteins. **e** Overall survival of all patients in relation to the combination of KAI1/CD82, CD44, MMP7 and β-catenin expression (log-rank = 52.882, *P* < 0.001). The green line represents positive expression of KAI1/CD82 and negative expression of CD44, MMP7, β-catenin and the blue line represents negative expression of KAI1/CD82 and positive expression of CD44, MMP7, β-catenin. The red line represents other positive or negative expression of the proteins. In all analyses, ┼ represents censored observation

In the univariate analysis, OS time was significantly correlated with clinicopathological factors, including depth of invasion (*P* = 0.005, log-rank = 7.781), lymph node metastasis (*P* < 0.001, log-rank = 24.335), distant metastasis (*P* = 0.003, log-rank = 8.818), TNM stage (*P* < 0.001, log-rank = 44.383) (Table 5). The increased expression of KAI1/CD82 had significant association with more favorable OS (*P* < 0.001, log-rank = 46.961) (Fig. 2a). In addition, overexpression of CD44, MMP7 and β-catenin predicted a poor prognosis in terms of OS time (log-rank = 24.611, 27.764, and 15.756, respectively; *P* < 0.001) (Fig. 2b,c and d). The combination of negative KAI1/CD82 expression and positive expression of CD44, MMP7 and β-catenin had a poorer prognosis compared with the contrary combination (log-rank = 52.882; *P* < 0.001) (Fig. 2e). Multivariate analysis revealed that expression of KAI1/CD82, MMP7 and β-catenin, and TNM stage were independent prognostic factors for OS (*P* < 0.05) (Table 6).

**Table 5:** Results of univariate analyses of overall survival (OS) time

Variables	*n*	Mean OS	*P*-value	Log-Rank
		(months)		
β-catenin			<0.001	15.756
Negative	45	66.1 ± 22.6		
Positive	129	46.8 ± 23.0		
KAI1/CD82			<0.001	46.961
Negative	118	42.9 ± 21.8		
Positive	56	70.5 ± 18.1		
CD44			<0.001	24.611
Negative	68	64.4 ± 22.3		
Positive	106	43.7 ± 22.2		
MMP7			<0.001	27.764
Negative	61	67.5 ± 20.3		
Positive	113	43.3 ± 22.1		
Sex			0.804	0.062
Male	101	53.7 ± 23.0		
Female	73	49.1 ± 26.0		
Age				0.057
60 years	68	52.7 ± 23.1	0.811	
≥ 60 years	106	51.2 ± 25.3		
Diameter of tumor			0.448	0.575
5.0 cm	104	50.9 ± 24.1		
≥ 5.0 cm	70	53.6 ± 24.8		
Location			0.217	1.521
Rectum	89	50.3 ± 25.7		
Colon	85	53.3 ± 22.9		
Differentiation			0.628	0.931
Well	39	55.7 ± 25.5		
Moderate	90	51.4 ± 23.9		
Poor	45	49.1 ± 24.4		
Depth of invasion			0.005	7.781
Under serous membrane	96	57.1 ± 23.9		
To serous membrane	78	45.2 ± 23.4		
Lymph node metastasis			<0.001	24.355
Negative	103	60.9 ± 20.3		
Positive	71	38.5 ± 23.7		
Distant metastasis			0.003	8.818
Negative	150	54.1 ± 24.1		
Positive	24	37.2 ± 21.0		
TNM stage			<0.001	52.689
I+ II	101	62.3 ± 19.7		
III + IV	73	37.2 ± 22.7

**Table 6:** Results of multivariate analyses of overall survival (OS) time

Covariate	Categories	Multivariate analysis		
		HR	95 % CI	*P*
TNM stage	I + II/III + IV	2.304	1.600to3.317	<0.001
β-catenin	Negative/positive	1.652	1.058to2.579	0.027
MMP7	Negative/positive	1.706	1.135to2.563	0.010
KAI1/CD82	Negative/positive	0.430	0.269to0.687	<0.001

## Discussion

Tumor metastasis is the major factor that restricts the prognosis of CRC. The progression of CRC is particularly associated with the mutation of various molecules, but few are used to predict metastasis in CRC. In this study, we analyzed the tumor metastasis related factors KAI1/CD82, CD44, MMP7 and β-catenin, to provide a new direction for investigating the metastasis and prognosis of CRC.

In present study, KAI1/CD82 protein expression was down-regulated in the progression of CRC [22]. In addition, KAI1/CD82 expression was significantly correlated with invasion, differentiation, TNM stage, distant metastasis and lymph node metastasis in CRC (Table 2). Muneyuki et al. and Maurer et al. reported that KAI1/CD82 expression decreased progressively with the advance of the tumor stage and was absent in lymph nodes [20, 23], which is consistent with our results (Table 2). Moreover, our data demonstrated that KAI1/CD82 expression decreased or was lost in CRC metastasis [20, 24], while Yang et al. indicated that KAI1/CD82 expression was regained in CRC associated with metastasis [25]. Furthermore, in our analysis, KAI1/CD82 expression was shown as a significant risk factor for lymph node metastasis (Table 4). Overall, although the KAI1/CD82 expression is still controversial in CRC progression, we conclude that KAI1/CD82 expression is significantly correlated with CRC metastasis.

Based on our analysis, there was no statistically significant relationship between CD44 expression and clinicopathological features such as age, sex, size, tumor location, differentiation, depth of invasion, which is consistent with some other reports [5, 26, 27] (Table 2). In addition, we found CD44 expression was significantly associated with TNM stage, lymph node metastasis and distant metastasis (Table 2). Moreover, in our study, the positive expression of CD44 was 74.6 % (53/71) in the lymph node metastasis group and 54.1 % (53/103) in the no lymph node metastasis tissues, which is in accordance with the results of Huh et al. [5]. Ropponen et al. also showed that CD44 expression was positively correlated with tumor stage [28]. Similar results were obtained in the present study, which indicated that CD44 overexpression is beneficial to CRC progression and metastasis.

MMP7 is a target of the Wnt/β-catenin pathway involved in multiple steps of CRC [29]. In this study, MMP7 protein expression was up-regulated in the progression of CRC [30]. Moreover, our study demonstrated that MMP-7 protein expression in CRC was positively associated with TNM stage, distant metastasis and lymph node status (Table 2), which is consistent with previous study [31], and indicates that MMP7 expression is closely related to CRC metastasis [21].

β-catenin is the key factor of the Wnt/β-catenin pathway, which is generally believed to be important for the development of CRC. Our results as well as those reported by Gao *et al*. indicate that positive β-catenin expression is significantly associated with TNM stage, distant metastasis and lymph node metastasis [19]. Moreover, in our analysis, β-catenin expression was shown as a significant risk factor for lymph node metastasis [32] (Table 4). In combination with our previous study, we demonstrate here that β-catenin plays an important role in CRC metastasis and prognosis [16, 33, 34].

As mentioned previously, some discrepancies were observed between our results and those of others. These differences could be due to the use of different antibodies, different IHC methods, as well as variation in patient material and analysis of the immunostaining. Nevertheless, we conclude that KAI1/CD82, CD44, MMP7 and β-catenin expression are related with metastasis of CRC. Moreover, increasing evidence demonstrates that cell surface adhesion and ECM components are crucial for tumor metastasis [35]. In particular, CD44, KAI1/CD82 and β-catenin are cell membrane proteins that bind to ECM or adhesion proteins [14, 36], and the MMP7 protein also plays a role in the degradation of the ECM at the cell surface [12]. In our study, we found that KAI1/CD82 expression was negatively correlated with β-catenin expression (Table 3). This is consistent with the study by Chigita et al. [37], in which KAI1/CD82 was shown to attenuate Wnt signaling by controlling the cellular distribution of β-catenin. Chairoungdua et al. [38] also demonstrated that KAI1/CD82 downregulated the Wnt signaling pathway through the exosomal discharge of β-catenin in human embryonic kidney 293 T cells (HEK 293 T cells). On the other hand, MMP7 and CD44 are considered to be target genes of the Wnt/β-catenin pathway [28, 39], which is supported by our observation of a positive correlation between β-catenin and the expression of CD44 and MMP7 (Table 3). From above analysis, we can see that KAI1/CD82, CD44 and MMP7 are related to Wnt/β-catenin pathway. In addition, based on our analysis, KAI1/CD82 expression was negatively correlated with CD44 and MMP7 expression (Table 3). Similarly, Wei et al. demonstrated that the ablation of KAI1/CD82 increased CD44 expression and enhanced migration and invasion in endothelial cells [40]. Furthermore, previous studies also demonstrated that KAI1/CD82 inhibited the activity of MMP2 [41] and MMP9 [42], which are reported to be activated by MMP7 [43]. Thus, we speculate that KAI1/CD82 may play a role in mediating the expression of CD44, MMP7 and β-catenin. Moreover, Yu et al. concluded that CD44 recruited MMP7 to the cell surface in a broad range of cell types [44], which is consistent with our analysis of the correlation (Table 3). Overall, these results indicate that there is a complex relationship between the KAI1/CD82, CD44, MMP7 and β-catenin in tumor progression. Combined with the results of the present study, we have reason to believe that the interaction of these factors is related to metastasis in CRC.

From our present study, we found that the tumor metastasis is closely related to the prognosis (Table 5). In accordance with other reports, our survival analysis showed that the reduction in KAI1/CD82 expression [24] and increasing CD44 [5, 28], MMP7 [30, 31], β-catenin [19] expression are indicators of a poor prognosis in CRC patients (Fig. 2). In multivariate analysis, KAI1/CD82 expression and MMP7 expression were identified as independent factors, which is consistent with the results of previous studies [20, 31] (Table 6), indicating that these molecules play important roles in CRC prognosis.

Collectively, although we used only IHC to investigate the relationship between these factors, and the number of specimens was relatively small, our results can still be considered to reflect the biological behavior of CRC metastasis. These results may represent the basis of a new method for predicting metastasis and a new foundation for the development of molecular therapy in CRC. In addition, this study provides a direction for future molecular and biochemical studies of CRC, particularly the relationship between these factors and the Wnt/β-catenin signaling pathway.

## Conclusion

In summary, low expression of KAI1/CD82 combined high expression of CD44, MMP7 and β-catenin was found to be associated with tumor metastasis and poor prognosis in CRC. Moreover,a correlation between these markers was also identified, and combined detection of these factors has potential for predicting metastasis and prognosis of CRC.

## Abbreviations

CRC: Colorectal carcinoma
MMP7: Matrix metalloproteinase 7
IHC: Immunohistochemical
TNM: Tumor-node-metastasis
OS: Overall survival
ECM: Extracellular matrix
CI: Confidence intervals
HR: Hazard ratio
